# In vitro assessment of Function Graded (FG) artificial Hip joint stem in terms of bone/cement stresses: 3D Finite Element (FE) study

**DOI:** 10.1186/1475-925X-12-5

**Published:** 2013-01-16

**Authors:** Fawzi F Al-Jassir, H Fouad, Othaman Y Alothman

**Affiliations:** 1 FRCSC, College of Medicine, King Saud University, Riyadh, Saudi Arabia; 2 Orthopedic Surgery Research Chair, King Saud University, Riyadh, Saudi Arabia; 3 Biomedical Engineering Department, Helwan University, Faculty of Engineering, Helwan, Egypt; 4 Department of Chemical Engineering, College of Engineering, King Saud University, Riyadh, Saudi Arabia

**Keywords:** Finite element, Hip joint, Stem length, Function graded material, Bone cement

## Abstract

**Background:**

Stress shielding in the cemented hip prosthesis occurs due to the mismatching in the mechanical properties of metallic stem and bone. This mismatching in properties is considered as one of the main reasons for implant loosening. Therefore, a new stem material in orthopedic surgery is still required. In the present study, 3D finite element modeling is used for evaluating the artificial hip joint stem that is made of Function Graded (FG) material in terms of joint stress distributions and stem length.

**Method:**

3D finite element models of different stems made of two types of FG materials and traditional stems made of Cobalt Chromium alloy (CoCrMo) and Titanium alloy (Ti) were developed using the ANSYS Code. The effects on the total artificial hip joint stresses (Shear stress and Von Mises stresses at bone cement, Von Mises stresses at bone and stem) due to using the proposed FG materials stems were investigated. The effects on the total artificial hip joint system stresses due to using different stem lengths were investigated.

**Results:**

Using FG stem (with low stiffness at stem distal end and high stiffness at its proximal end) resulted in a significant reduction in shear stress at the bone cement/stem interface. Also, the Von Mises stresses at the bone cement and stem decrease significantly when using FG material instead of CoCrMo and Ti alloy. The stresses’ distribution along the bone cement length when using FG material was found to be more uniform along the whole bone cement compared with other stem materials. These more uniform stresses will help in the reduction of the artificial hip joint loosening rate and improve its short and long term performance.

**Conclusion:**

FE results showed that using FG stem increases the resultant stresses at the femur bone (reduces stress shielding) compared to metallic stem. The results showed that the stem length has significant effects on the resultant shear and Von Mises stresses at bone, stem and bone cement for all types of stem materials.

## Introduction

It is well known that the fully understanding of stresses’ distribution in the hip joint is useful for both pre-operative planning and post operative rehabilitation. The short and long term behavior of the total hip joint replacement is dependent on obtaining optimal stresses’ distribution within the bone implant construct [1-3]. The structure, shape and material are the three main factors considered in the design of the hip prosthesis [4-7]. Initially, the artificial hip joint implants are used in orthopedic surgeries without prior pre-clinical testing that may lead to unsatisfactory clinical results. The pre-clinical testing helps in improving the clinical performance of the total hip joint replacement and limiting the possibility of joint revision operations [8].

The previous results indicated that if the design of hip joint stem resulted in high stresses in the fixation areas of the prosthesis, the joint fracture in short term or fatigue fracture in long term is quite likely to occur [9,10]. Also, the presence of non uniform stresses’ distribution around the implanted stem will result in changes in bone density and shape [11]. These non-uniform stresses’ distribution can cause the fixation to be altered as the bone density changes. For these reasons, many experimental and analytical studies have been implemented to optimize the stresses distribution in total hip joint replacement through the modification of stem design parameters [12-16]. These results indicated that the bone cement material, stem shape and material have great effects on the total hip joint short and long term performance. Chenm WP et al. [17] investigated the roles of bone cement in different fixation configurations of total hip arthroplasty initial stress shielding. Three different configurations of cement fixation: cement less, proximally-cemented and fully-cemented fixations were used in their study. The results revealed that, less stress shielding was found for femur with fully-cemented fixation. Takafumi U. et al. [18] studied the effects of bone cement type on the resulting stress shielding on the total hip joint replacement. Two types of bone cement; i.e. PMMA and Bioactive bone cement, were used. The results indicated that some bone resorption was observed in the use of bioactive bone cement 24 months after operation. However bone resorption was not observed 24 months after operation in the use of PMMA. Sabatini and Goswami [11] investigated the effect of stem cross section and material on the resultant Von Mises stresses on the stem designated locations. Stainless Steel (SS316L), Cobalt Chromium alloy (CrCoMo) and Titanium alloy (Ti 6Al 4 V) were used as stem materials. The results indicated that using high modulus stem created high stresses at the implant distal end while using low modulus stem created high stresses at the implant proximal end. These high stresses are known to affect the short and long term performance of the artificial hip joint.

As can be seen, the hip joint stem is usually made of SS, CrCoMo and Ti alloys. These materials have high modulus compared to the surrounding bone. This modulus mismatching is one of the main reasons for joint loosening due to the changes in the surrounding bone density (stress shielding). Therefore, the need for special stem material with suitable mechanical properties is essential for improving the performance of the total joint replacement. It is known that the Function Graded Material (FGM) can provide a reasonable compromise in terms of the properties of materials that would not be achieved otherwise. This is because the microstructure of the FGM is inhomogeneous and changing continuously in space. In comparison to conventional composite materials, FGMs exhibit a progressive change in composition, structure, and properties as a function of position within the material. With tailored design, the mechanical performance of FGMs can be superior to that of the conventional composite with a uniform composition. Wide varieties of available processes have been reported for FGM fabrication, such as plasma spraying, powder metallurgy, physical vapor deposition (PVD), and so on [19,20].

Hedia HS et al. [21,22] studied the effect of using 2D-FG Material cement less hip stem material in the reduction of bone resorption around cement less hip implants. The results indicated that the recommended FG stem reduced the stress shielding and reduced the maximum interface shear stress at the lateral and medial sides of the femur in comparison with Ti stem. Gong H. et al.[23] identified the effects of using FG materials as cement less femoral stem on the functional adaptive behaviors of bone. The results showed that 2D-FG Material stem might produce more mechanical stimuli and more uniform interface shear stress compared with the stems made of other materials. Oshkour AA. et al. [24] developed 3D FE model of a FG femoral prosthesis. The model consisted of FG femoral prosthesis, bone cement, and femur. The results indicated that using FG stem resulted in uniform stress on the cement mantle layer and reduction of stress shielding in the joint.

Although FGMs have a wide range of applications from aviation structures to computer components and a promising potential in the biomedical usage, the use of FGMs is limited in the field of femoral prostheses. As such, the leading objective of this study was to develop new stem using FGMs in such a way that they reduce stress shielding on the hip prosthesis. This study intended to investigate the effects of using longitudinal FG material stem with low modulus at distal end and high modulus at proximal end in the improvement of the total hip joint replacement performance. In this work, the 3D FEM is used for investigating the modification on the hip joint stresses when using FG material as stem instead of traditional stems made of Ti and CoCrMo under static loading conditions. Also, the effects of stem length on the resultant stresses at the hip joint component will be considered.

### Finite element model

**Figure 1:**
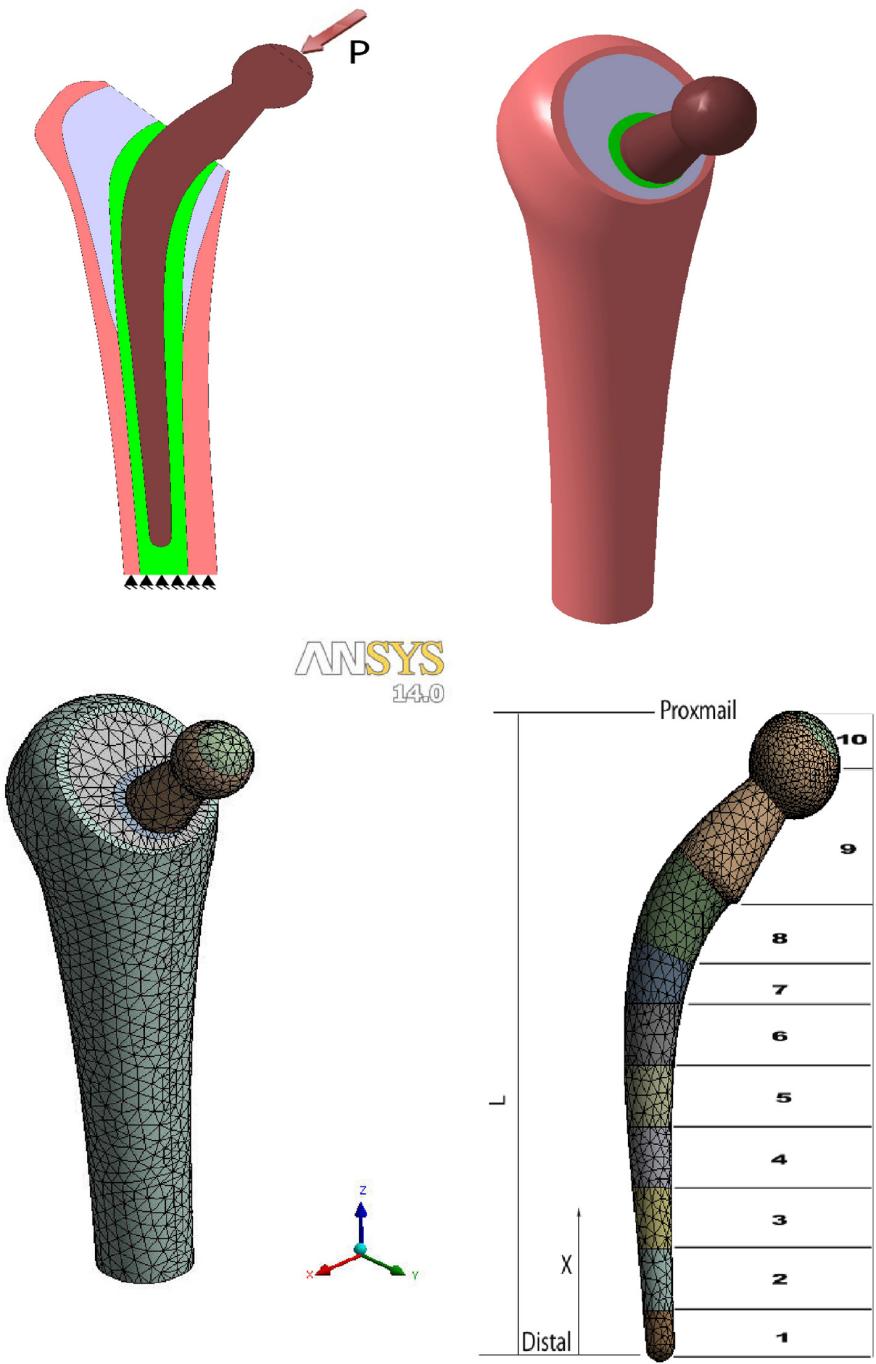
Hip joint geometry and loading conditions.

In the present work, the different stem geometries definitions, boundary conditions, loads and solution are all performed using the FE code ANSYS v14 [25]. This implicit Lagrangian non-linear finite element code is used because of its efficiency for linear and non-linear quasi-static simulation. The geometries of partial disassemblies’ configurations and combination of mapped and free meshing used to generate the hip joint models are shown in Figure 1. Four different stem lengths will be considered in this study (135 mm, 165 mm, 190 mm and 220 mm). The femur length is assumed to have a constant length for all stems while the bone cement is totally surrounding the stem and filling a constant length of the medullary cavity (35 mm) for all FE models. The dimensions of stem are taken from real Charnley model that has 165 mm length. The other stems dimensions (135, 190 and 220 mm length) were modified from the original stem (165 mm length). Only the length has been changed. The dimensional geometries of the hip joint are constructed with CATIA [25] and then imported to the ANSYS code. A 3D 20 node solid element (tetrahedron Solid 95) is chosen for modeling the hip joint assembly. This element has the capability to accurately model the plastic deformation, large deflection and large strain that may occur during the simulation. Also Tetrahedron Solid 95 is chosen due to its ability to model regular and irregular shapes

### Assigning materials properties to the FEM

In the present study four different types of materials were used to simulate the stem behavior in the FE code. These materials are Ti alloy, CoCrMo and two longitudinal FG materials. The first FG material is assumed to have Young’s modulus of 220 GPa at the proximal end that decreases exponentially to 50 GPa at the distal end. The second FG material is assumed to have Young’s modulus of 100 GPa at the proximal end that decreases exponentially to 10GP at the distal end. All the FG materials are assumed to have a constant Poisson’s ratio of 0.3 [26-28]. The functional relationship that represents the variation of stem Young’s modulus along its length is given in the following equation [28].

(1) $$E_{X}=\;E_{d}\;e^{-\ln\varphi },$$

where $\varphi ={\left(\frac{E_{d}}{E_{p}}\right)}^{\frac{X}{L}}$

*E*_*x *_is the Young’s modulus of the FG stem at any length, *E*_*d *_is the value of stem modulus at the distal end, *E*_*p *_is the value of stem modulus at the proximal end, L is the total length of the stem and X is the distance from the distal end towards the proximal end. It is evident that at X= 0, the value of *E*_*x *_equals *E*_*d *_and when X= L, the value of *E*_*x *_equals *E*_*p*_.

The Ti alloy stem is assumed to have a uniform modulus of 110 GPa and Poisson’s ratio of 0.3 [26-28]. The CoCrMo stem is assumed to have uniform modulus of 220 GPa and Poisson’s ratio of 0.3 [10,28]. The femur bone is assumed to be composed of cancellous and cortical bones (Figure 1). The cortical and cancellous bones are assumed to have Poisson’s ratio of 0.3 and uniform modulus of 17 GPa and 3 GPa respectively [12,15,28]. Finally, the PMMA bone cement is assumed to have a uniform modulus of 2.5 GPa and Poisson’s ratio of 0.38 [15]. The mechanical properties of stem, bone and PMMA bone cement are also tabulated in Table 1. All the materials in the FE simulation are assumed to be isotropic and linearly elastic. In the FEM, the FG stems are divided into 10 parts with different modulus which varies with stem length according to Equ.1 (Figure 1). The modulus of each part of stem is assumed to be isotropic and linearly elastic. Table 2 shows the variation of FG stem modulus with stem length used in the FE model.

**Table 1:** Mechanical properties of the materials used in the hip joint model

	**Ti alloy**	**Co Cr Mo**	**Cancellous bone**	**Cortical bone**	**Bone cement**	**FG1**	**FG2**
**Elastic Modulus GPa**	110	220	3	17	2.5	220 to 50 GPa	110-to 10 GPa
**Poisson’s Ratio**	0.3	0.3	0.3	0.3	0.38	0.3	0.3

**Table 2:** Variation of FG stem modulus with stem length that used in the FE model

**FG stem part No**		**1**	**2**	**3**	**4**	**5**	**6**	**7**	**8**	**9**	**10**
**Modulus (GPa)**	**FG1**	10	12.7	16.1	26	33	42	53	71	104	110
	**FG2**	50	58	77	90	105	121	141	169	213	220

The effects of mesh size and density on the predicted results are examined by increasing the number of elements until the predicted results become constant with increasing the mesh density. The contact interfaces between bone, bone cement and stem are represented in the finite element simulation as completely bonded surfaces. Also, the distal end of the femur is assumed to be completely fixed in all directions. The Finite Element Model loading conditions and constrains are shown in Figure 1.

The loading of the hip joint is based on the assumption that the patient body weight is 70Kg. The other initial conditions such as sex, age, activity etc. are neglected. This weight results in an equivalent hip joint load of 3KN which is applied as uniform pressure normal to the implant femur head [11] (Figure 1). This resultant load can be attributed to the fact that the typical gait human cycle generates forces in hip joint ranging from 4–6 times the body weight [14,28].

## Results and discussion

### Effects of stem length on the joint stresses

#### Von Mises stresses at bone cement

**Figure 2:**
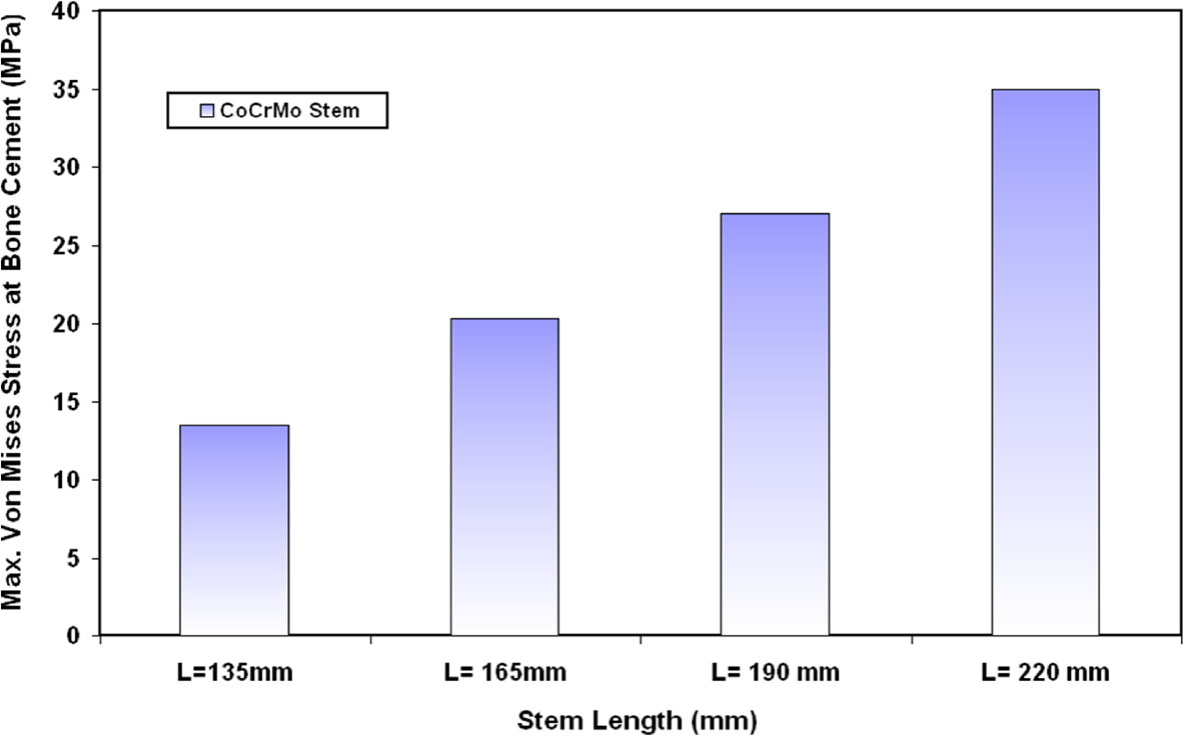
Variations on the maximum Von Mises stresses at the bone cement for CoCrMo stems with different lengths.

**Figure 3:**
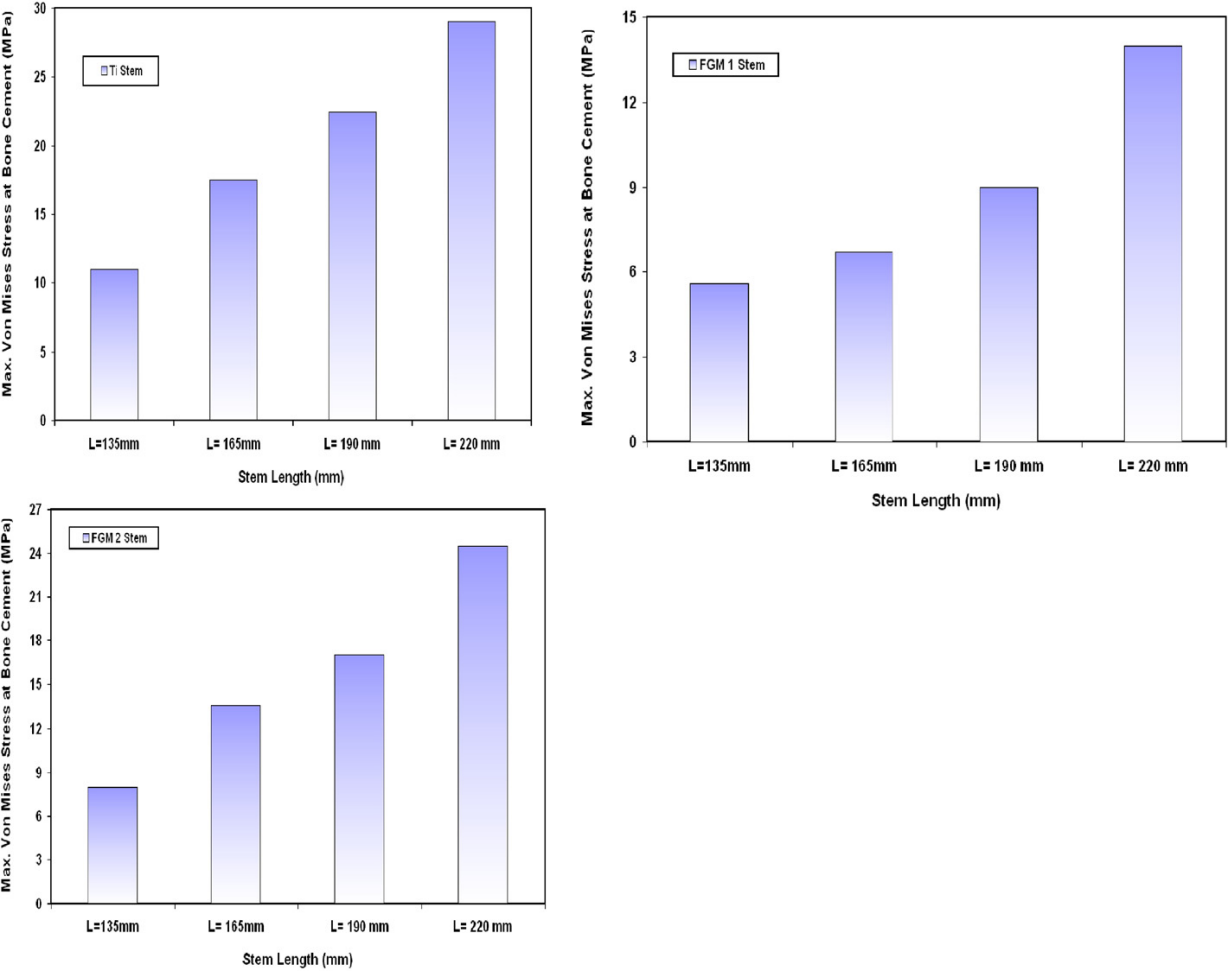
Variations of maximum Von Mises stresses at the bone cement for Ti alloy, FG1 and FG2 stems with different lengths.

Figure 2 shows the variations on the maximum Von Mises stresses at the bone cement when using CoCrMo stems with different lengths. The model analysis of hip joint stresses shows that the greatest extreme values of stresses take place around the stem end (distal point). It can be remarked that the maximum Von Mises stresses at the bone cement increase significantly when increasing the stem length. For example, the stress at the bone cement increases by 50 % when a stem of 190 mm length is used instead of a 135 mm stem length. The significant increase in bone cement stress due to using long stems can be attributed to the increase in the distance between point of load application (at the femur head) and the hip joint fixation point (at the distal end). The longer distance between the loading point (proximal end) and the hip joint fixation point (distal end) induce larger bending stresses at the distal end. For CoCrMo stem, the values of maximum Von Mises stresses on the bone cement are 13.5 MPa, 20.3 MPa, 27 MPa and 35 MPa for 135 mm, 165 mm, 190 mm and 220 mm stem length respectively. These values are lower than the yield strength of bone cement (PMMA) which is around 39 MPa [29]. Similar trends have been also obtained for the variations of maximum Von Mises stresses at the bone cement when using Ti alloy, FG1 and FG2 stems with different lengths as indicated in Figure 3.

**Figure 4:**
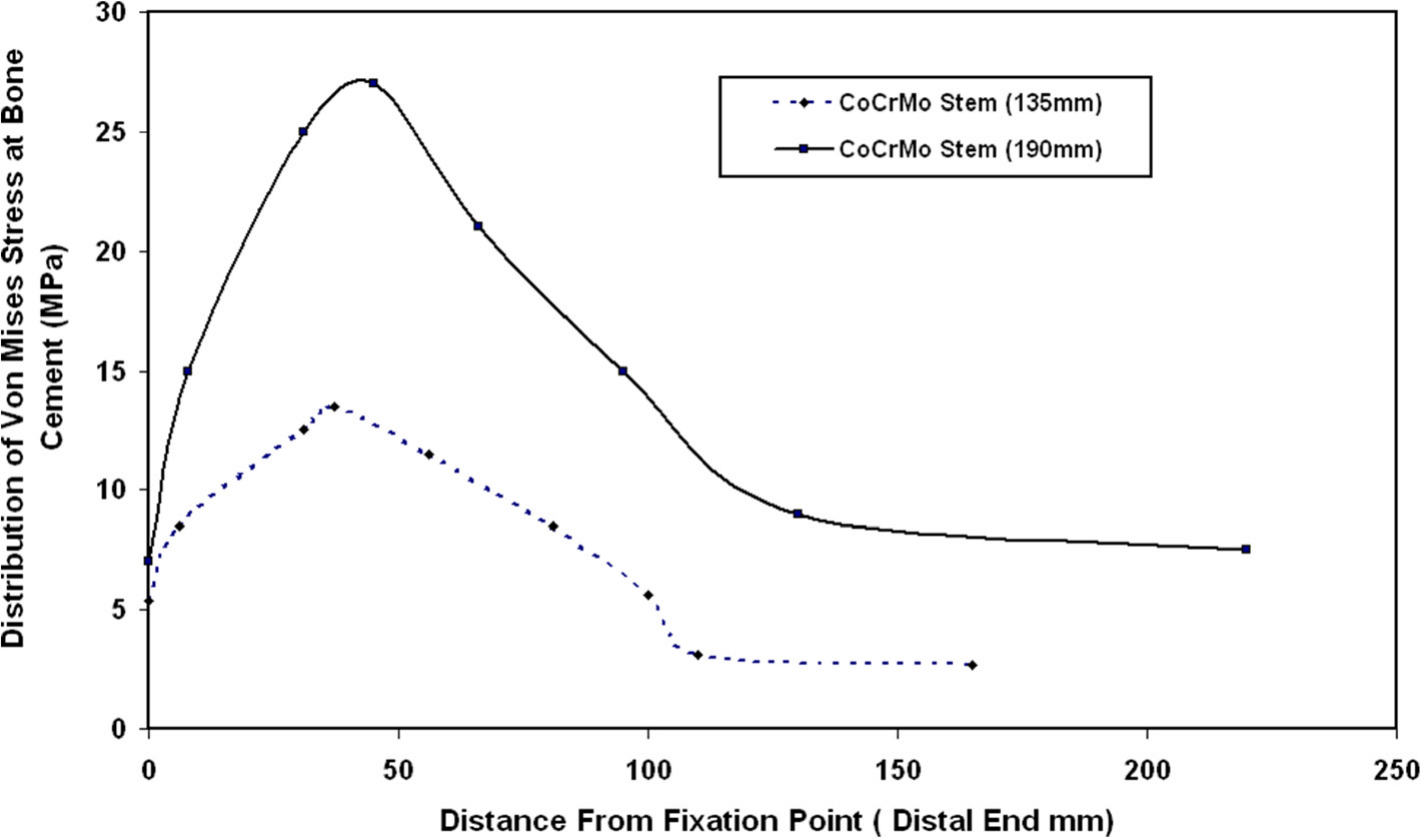
Distribution of maximum Von Mises stresses at bone cement along the cement length for 135 mm and 190 mm CoCrMo stems.

Figure 4 shows the distribution of maximum Von Mises stresses at bone cement along the cement length for 135 mm and 190 mm CoCrMo stems. The results of this figure indicate that the maximum Von Mises stress at bone cement takes place at the distal end (end of stem with bone cement). The values of these stresses increase as the stem length increases and their minimum values are observed at the proximal end of the hip joint. There is no change in the stresses’ distribution profile due to using different stem lengths, only the value of maximum Von Mises stresses increases when increasing the stem length.

### Shear stresses at bone cement/stem interface

**Figure 5:**
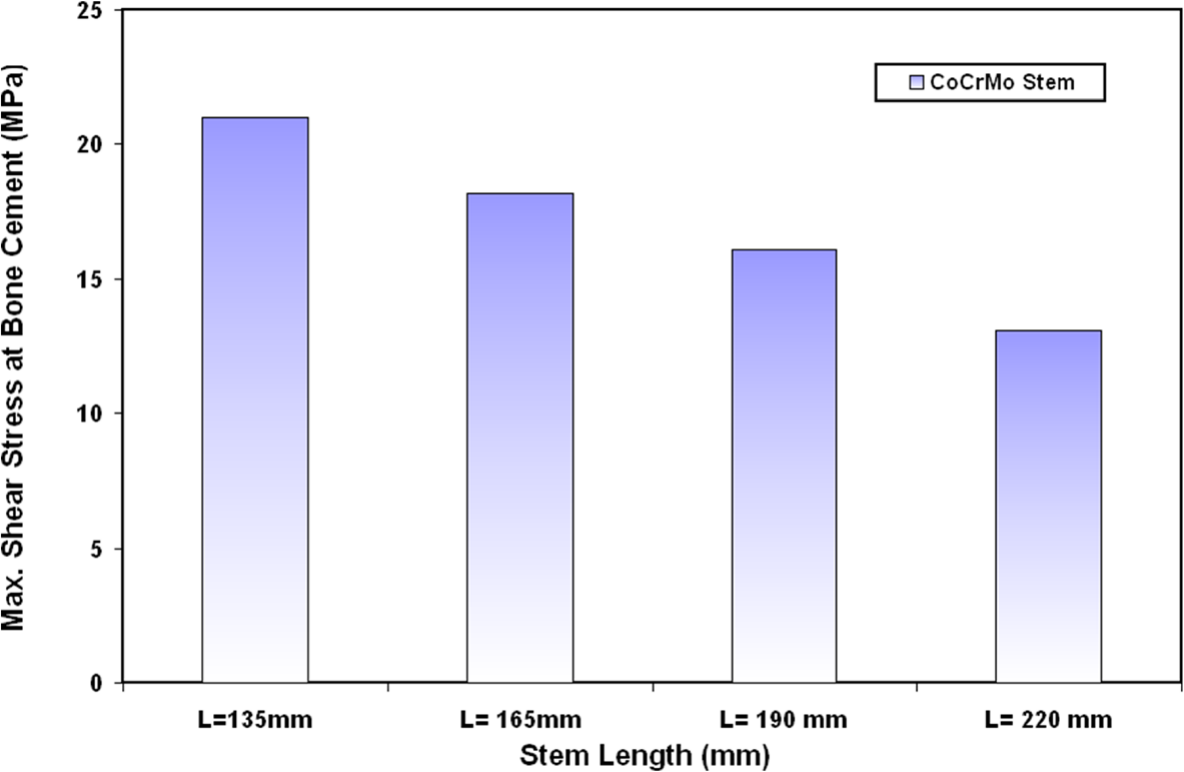
Variations on the maximum shear stresses between the bone cement and CoCrMo stems with different lengths.

**Figure 6:**
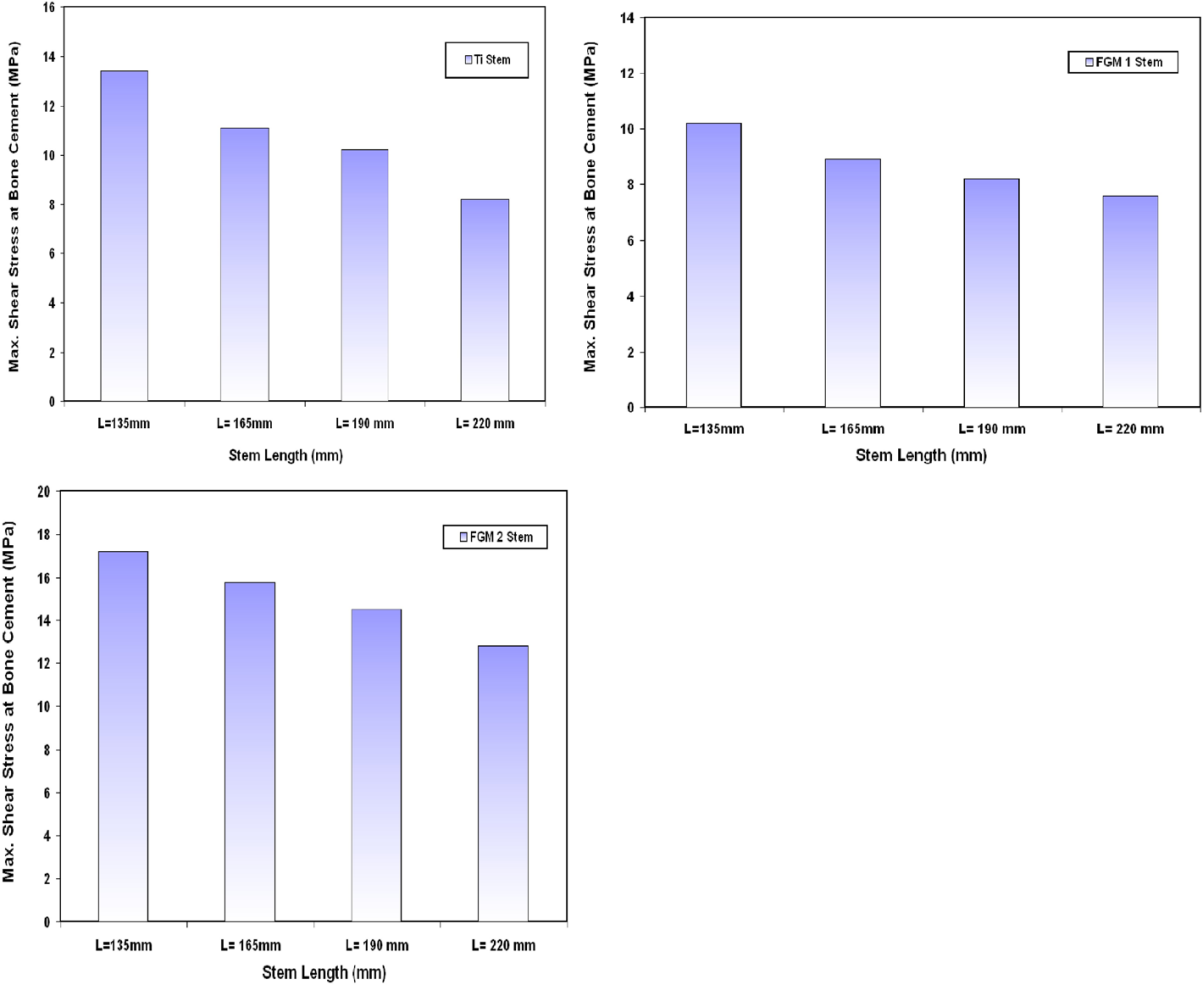
Variations of maximum shear stresses at the bone cement for Ti alloy, FG1 and FG2 stems with different lengths.

Figure 5 shows the variations on the maximum shear stresses at bone cement and CoCrMo stem interface for different stem lengths. The results indicate that the stem length has remarkable effect on the resultant shear stresses at the bone cement/stem interface. These stresses decrease when increasing the stem length. The maximum values of stresses are located at the distal end of the prosthesis as observed in many previous results [13,30]. The results indicate that the shear stress at the bone cement decreases by 38%, 23.3% and 13.3% when a stem of 220 mm,190 mm and 165 mm length is used instead of 135 mm stem length respectively. Similar trends have been also obtained for the variations of maximum shear stresses at the bone cement when using Ti alloy, FG1 and FG2 stems with different lengths as indicated in Figure 6. The values of maximum shear stresses on the bone cement are 21 MPa, 18.2 MPa, 16.1 MPa and 13.1 MPa for 135 mm, 165 mm, 190 mm and 220 mm CoCrMo stem length respectively. Similar results have been obtained by Sivasankar M. et al. [31] for the variation on shear stresses at the stem/cement interface with stem length. In this study, stem lengths of 145, 147.5, 150 and 155 mm are used in their finite element modeling. The shear stresses at the interface between bone cement and different stems are found to be related to the stem length. The maximum shear stresses at bone cement are found to be changed from 21 MPa to 17.1 MPa when 147.5 mm stem is used instead of 152.5 mm. This reduction can be attributed to the increase of contact area between bone cement and stem when using long stems.

### Von Mises stresses at bone

**Figure 7:**
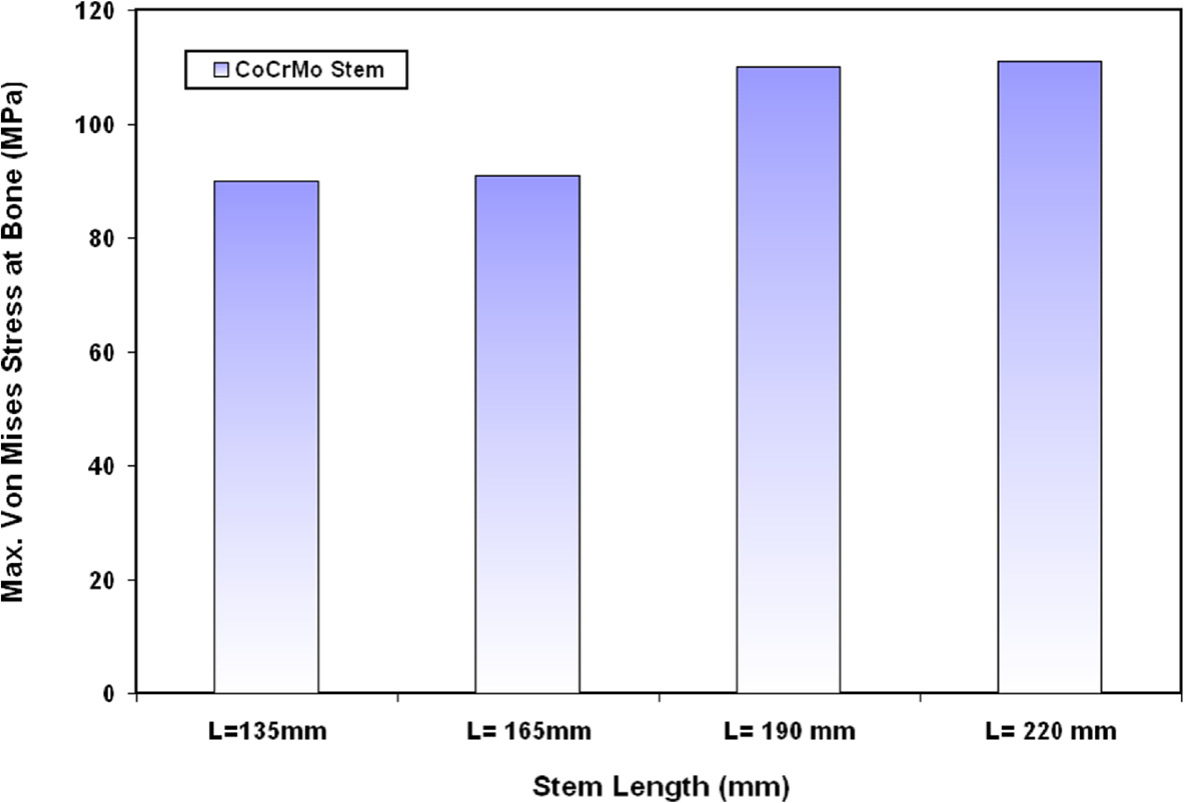
Variations of maximum Von Mises stresses at the bone for different CoCrMo stems.

**Figure 8:**
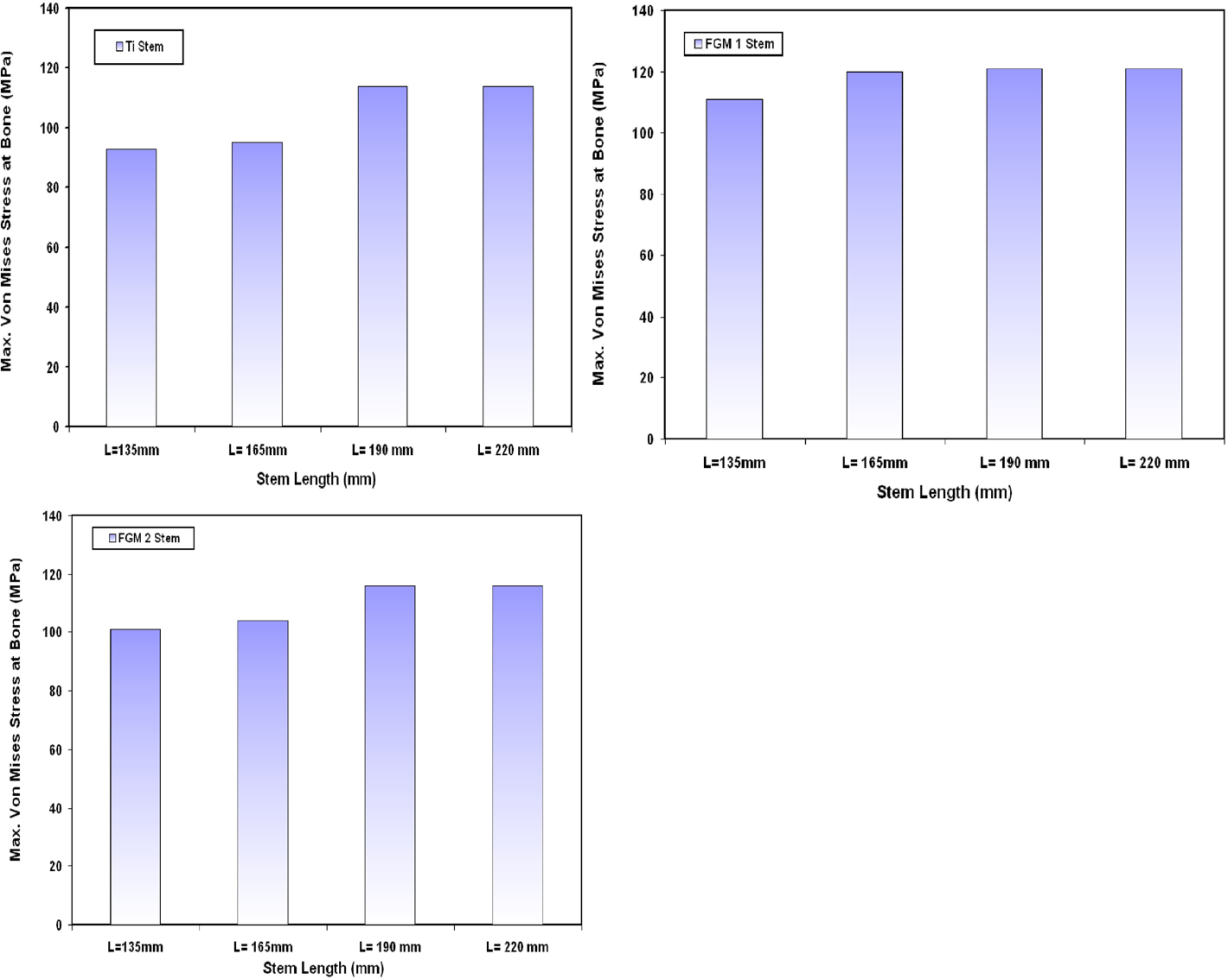
Variations of maximum Von Mises stresses at the bone for Ti alloy, FG1 and FG2 stems with different lengths.

The variations of maximum Von Mises stresses at the bone for different CoCrMo stems are shown in Figure 7. The results indicate that the resultant Von Mises stress at bone slightly increase with increasing the stem length. The greatest extreme values of bone stresses take place around fixation point of the stem. The results show that the stresses at bone increase by 1.3%, 22% and 23.3 % when a CoCrMo stem of 165 mm, 190 mm and 220 mm length is used instead of 135 mm CoCrMo stem respectively. Similar trends have also been obtained for the variations of maximum Von Mises stresses at the bone when using Ti alloy, FG1 and FG2 stems with different lengths as indicated in Figure 8. The values of maximum Von Mises stresses on the bone are 111 MPa, 120 MPa, 120 MPa and 121 MPa for 135 mm, 165 mm, 190 mm and 220 mm FG1 stems respectively. Similar results have been obtained by Abdullah A.H. et al. [32] where the stresses at bone increased from 112 MPa to 204 MPa and to 278 MPa when a long stem is used instead of medium and short length stem. It is important to mention that in the present study the maximum stresses at bone are below the yield strength of bone that ranges from 130 MPa to 160 MPa even for long stem [11,33].

**Figure 9:**
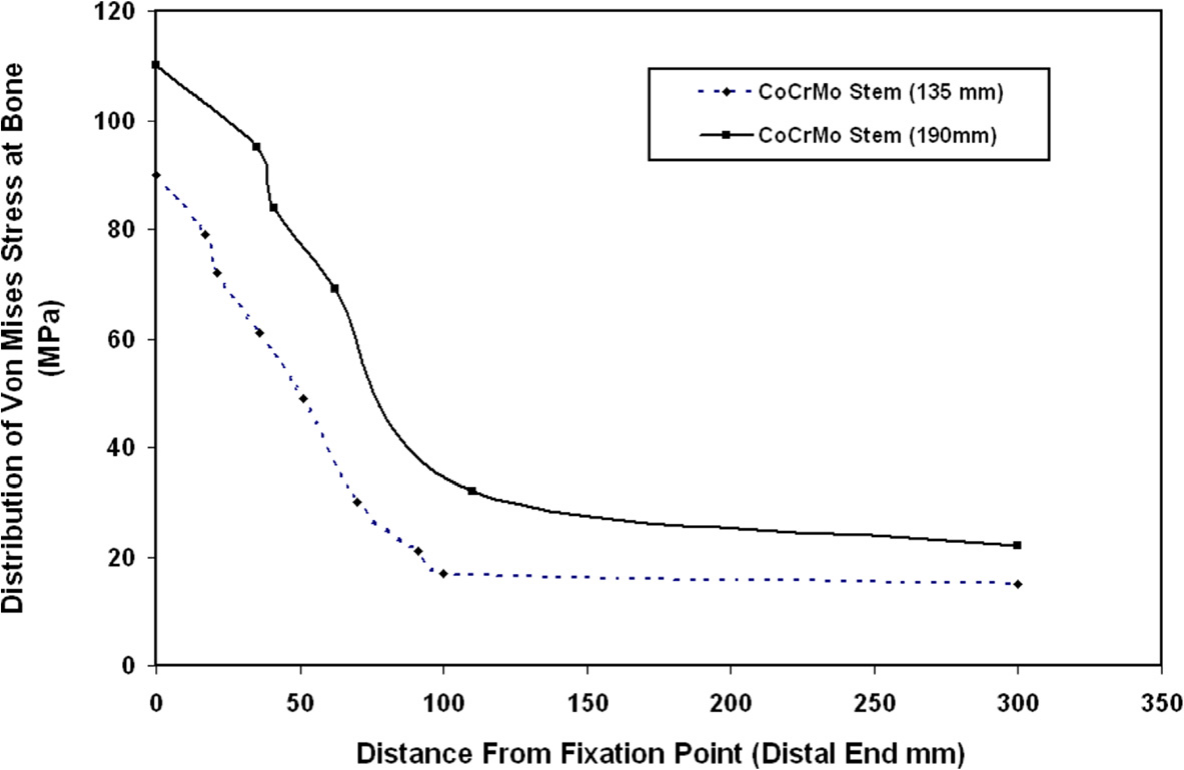
Distribution of Von Mises stresses at bone along the bone length for 135 mm and 190 mm CoCrMo stems.

Figure 9 shows the distribution of Von Mises stresses at bone along the bone length for 135 mm and 190 mm CoCrMo stems. The results of this figure indicate that the resultant Von Mises stresses at bone increase when increasing the stem length. These stresses decrease from their maximum value at the femur fixation point to their minimum values at the proximal end. According to the present FE results, the use of longer stems is believed to give higher stresses at femur and then be capable of reducing stress shielding problems while the use of shorter stems decreases the load transfer to the cortical bone and encourages the stress shielding, bone resorption problems and then prosthesis fails.

### Effects of stem material on the joint stresses

#### Von Mises stresses at bone cement

**Figure 10:**
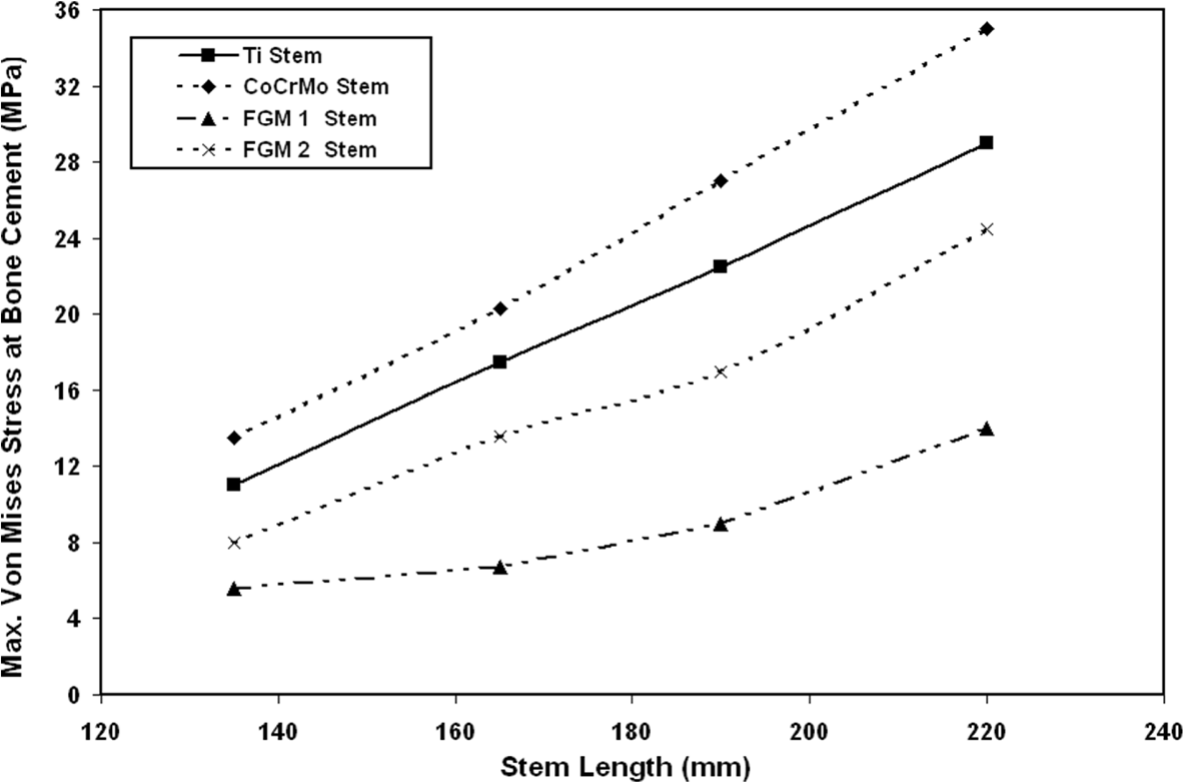
Effects of stem stiffness on the variations of maximum Von Mises stresses at the bone cement.

The effects of stem stiffness on the variations of maximum Von Mises stresses at the bone cement are shown in Figure 10. The results indicate that the maximum value of stresses occurs at the stem end/cement interface. From this figure, it can be noticed that the resultant Von Mises stresses at the bone cement decrease significantly when using FG materials instead of CoCrMo or Ti ally. For 135 mm stem, the Von Mises stresses at the bone cement decrease by 30%, 49% and 58.5% when using FG1 instead of FG2, Ti alloy and CoCrMo stem respectively. For 190 mm stem length, the Von Mises stresses at the bone cement decrease by 47%, 60% and 66.7% when using FG1 material instead of FG2, Ti alloy and CoCrMo stem respectively. For 220 mm stem length, the maximum Von Mises stresses at bone cement decrease from 35 MPa to 14 MPa when using FG1 stems instead of CoCrMo stem.

From the above results, it can be concluded that the proposed FG stem results in significant reduction in the resultant Von Mises stresses at bone cement compared with Ti and CoCrMo stems. The significant decrease in bone cement stress due to using FG stems can be attributed to the fact that low stiffness FG material allows for more deformation and can absorb more strain energy compared to high stiffness ones. The resultant maximum Von Mises stresses induced in bone cement when using FG1 (14 MPa) is much lower than the yield strength of bone cement (PMMA), which is around 39 MPa [29]. Similar trends are obtained by Senalp et al.[10], where the resultant stresses on the artificial hip joint decrease when using Ti alloy stem instead of CoCrMo stems under static and dynamic loading conditions. Also, the results obtained by Sabatani et al. [11] indicate that the use of Ti6Al4V stem exhibits lower stresses than those obtained when using CoCrMo and SS stems. Finally, Rawal et al. [33] indicated that the use of stem material with lower modulus (E=117 GPa) would be more beneficial for artificial hip joint performance than the use of stiffer material such as steel (E=210 GPa).

**Figure 11:**
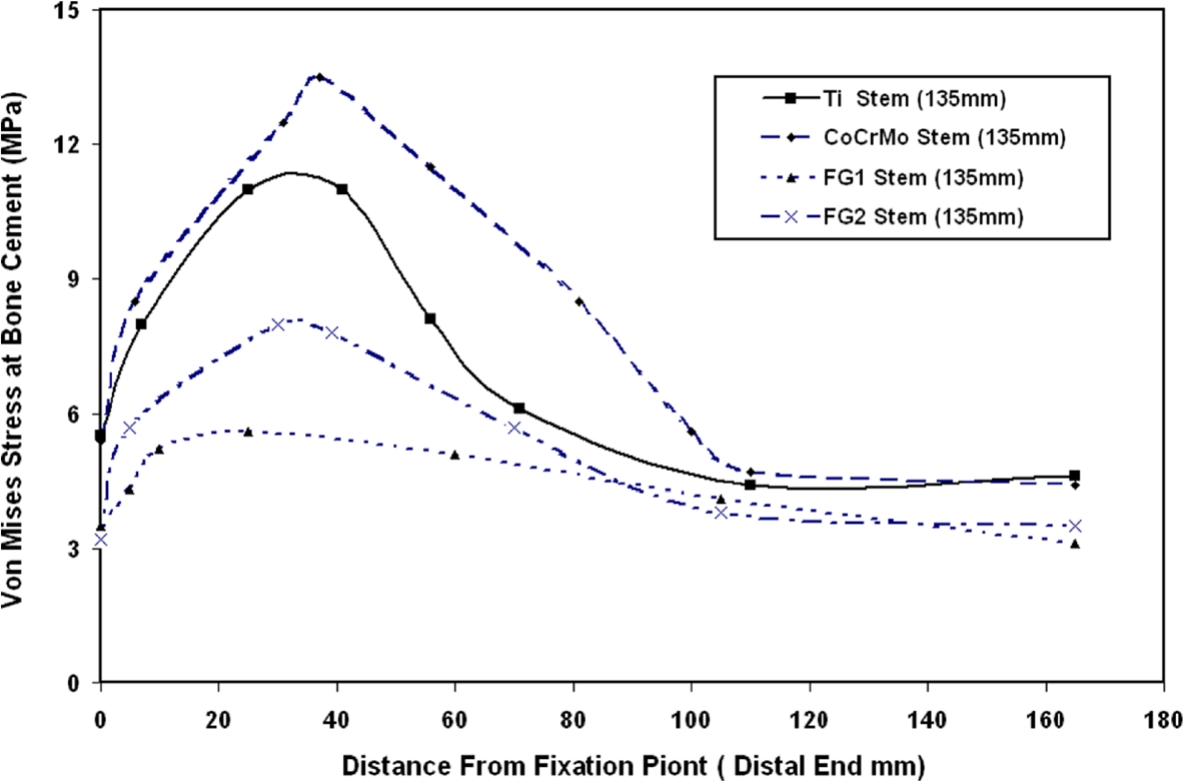
Distribution of maximum Von Mises stresses at bone cement along the cement length for 135 mm FG1, FG2, Ti alloy and CoCrMo stems.

Figure 11 shows the distribution of maximum Von Mises stresses at bone cement along the cement length for 135 mm FG1, FG2, Ti alloy and CoCrMo stems. The results of this figure indicate that the maximum Von Mises stress at bone cement takes place at the joint distal end. From the results, it can be concluded that the use of proposed FG1 as stem successfully reduces the predicted stresses at bone cement. Also, the stresses’ distribution along the bone cement length when using FG1 is more uniform along the whole bone cement compared to other stem materials. The more uniform predicted stresses at the bone cement that induced when using FG1 will help in the reduction of the artificial hip joint loosening rate and improving its short and long term performance.

### Shear stresses at bone cement/stem interface

**Figure 12:**
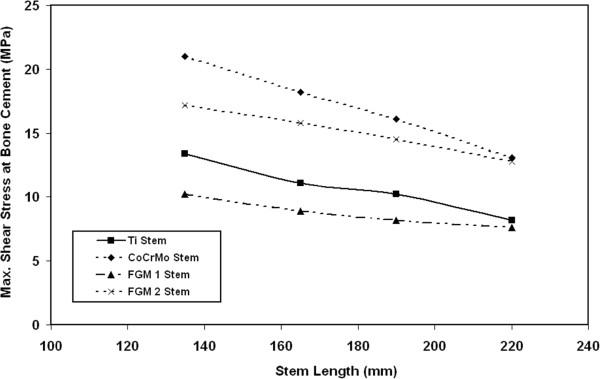
Variations on the maximum shear stresses at the bone cement with stem length.

Figure 12 shows the variations on the maximum shear stresses at the bone cement/stem interface with stem stiffness. The results indicate that the maximum value of stresses occurs at the distal end. It can also be noticed that the resultant shear stresses at the bone cement decrease significantly when using FG1 material instead of CoCrMo, Ti ally and FG2 even for long stems. The decrease in shear stress at bone cement/stem interface can be attributed to the low stiffness of FG stem compared with other stems. For example, for 135 mm stem, the shear stresses at the bone cement decrease from 13.1 MPa, 12.8 MPa, 8.2 MPa to 6.7 MPa when using FG1 material instead of CoCrMo, Ti alloy and FG2stem respectively. For 190 mm stem length, the shear stresses at the bone cement decrease by 51%, 44% and 12% when using FG1 material instead of CoCrMo, FG2 and Ti alloy stem respectively. For 220 mm stem length, the maximum shear stresses at bone cement decrease from 21 MPa to 10.2 MPa when using FG1 stems instead of CoCrMo stem. The significant decrease in bone cement shear stress due to using the proposed FG material even for long stems will improve the short and long term performance of the total hip joint replacement. It can be concluded that in order to obtain acceptable and uniform stresses at the bone cement, a stem of suitable length (not too long or too short) and low stiffness is required.

### Von Mises stresses at bone

**Figure 13:**
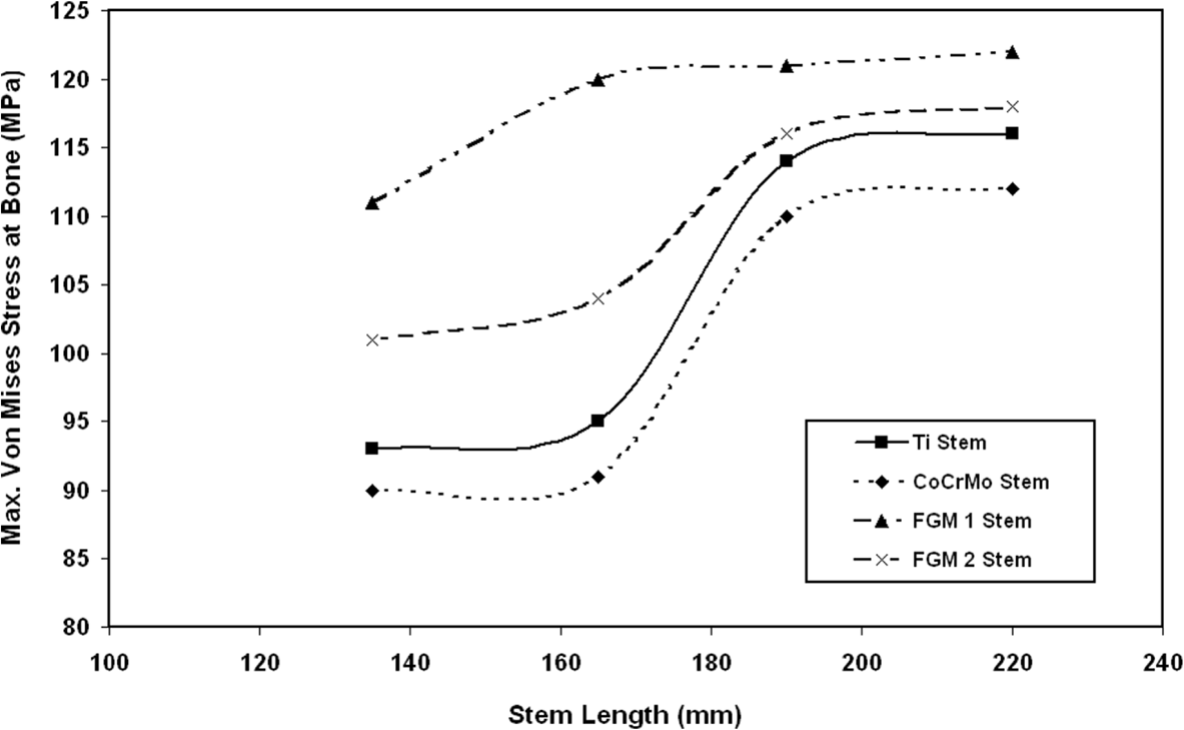
Effects of using FG material on the variations of maximum Von Mises stresses at the bone compared to other stem materials.

The effects of using the proposed FG stem on the variations of maximum Von Mises stresses at the bone compared to other stem materials are shown in Figure 13. The results indicate that using FG stem results in creating higher values of Von Mises stress at bone for all stem lengths. Also, the results indicate that the stresses at bone increase by 3.3%, and 23.3% when a 135 mm FG1 stem is used instead of 135 mm Ti alloy or CoCrMo stems. The maximum value of Von Mises stresses at bone when using the proposed FG material ranges from 111 MPa to 122 MPa. These stresses are below the yield stress of bone with factor of safety 1.6, indicating no risk of fracture [11,33]. The increase in bone stresses due to using FG material can be attributed to high flexibility of hip joint structure when using FG1 stem. The flexibility of hip joint structure in bending is known to be mainly determined by the properties of femur bone (E= 17 GPa) and stem (10 GPa at distal end). This means that the low stiffness (FG material) stem will increase the stresses at the femur bone (reduce stress shielding) compared to high stiffness stems (Ti and CoCrMo).

**Figure 14:**
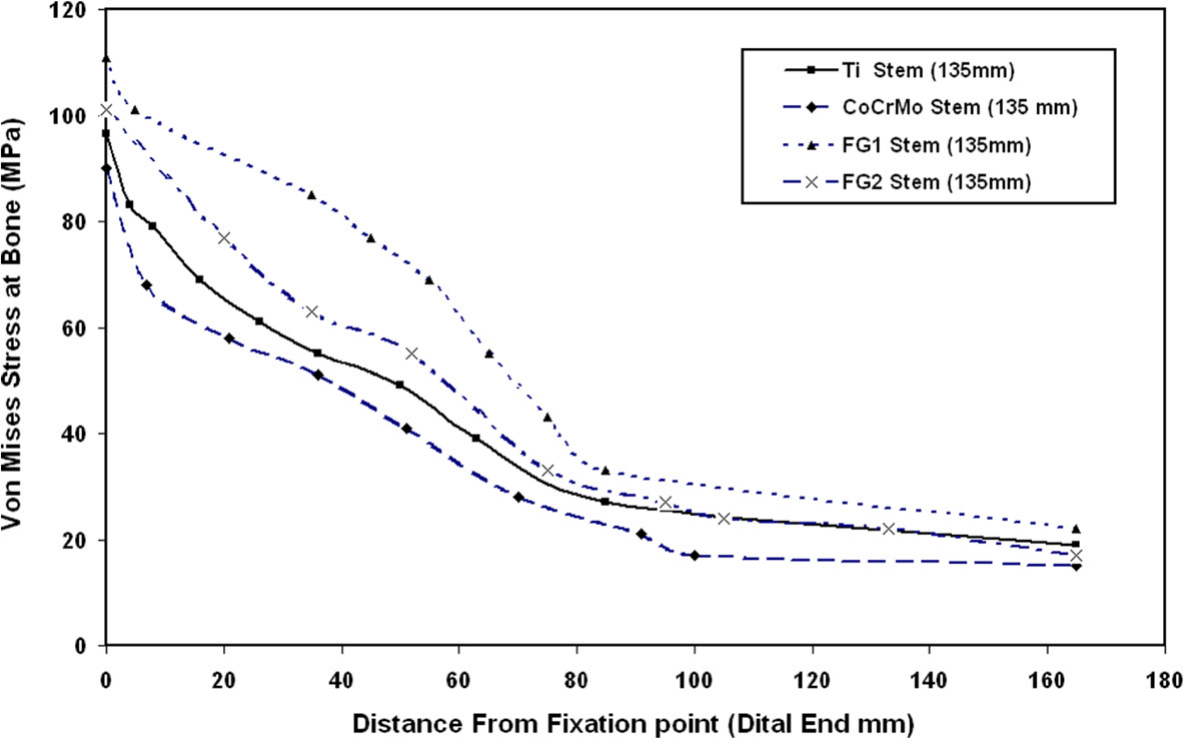
Distribution of Von Mises stresses at bone along the bone length for 135 mm FG1, FG2, Ti alloy and CoCrMo stems.

Figure 14 shows the distribution of Von Mises stresses at bone along the bone length for 135 mm FG1, FG2, Ti alloy and CoCrMo stems. The results of this figure indicate that the level of Von Mises stresses at bone increases on the whole femur when using FG stems compared to Ti alloy and CoCrMo stems. These stresses decrease from their maximum values at the femur fixation point to minimum values at the proximal end. The minimum values of stresses at bone increase by 47% when using FG1 stem instead of CoCrMo stem. Therefore, according to the present FE results, the use of FG material is believed to give higher stresses at whole femur bone and then capable of reducing stress shielding problem.

### Stresses at stem

**Figure 15:**
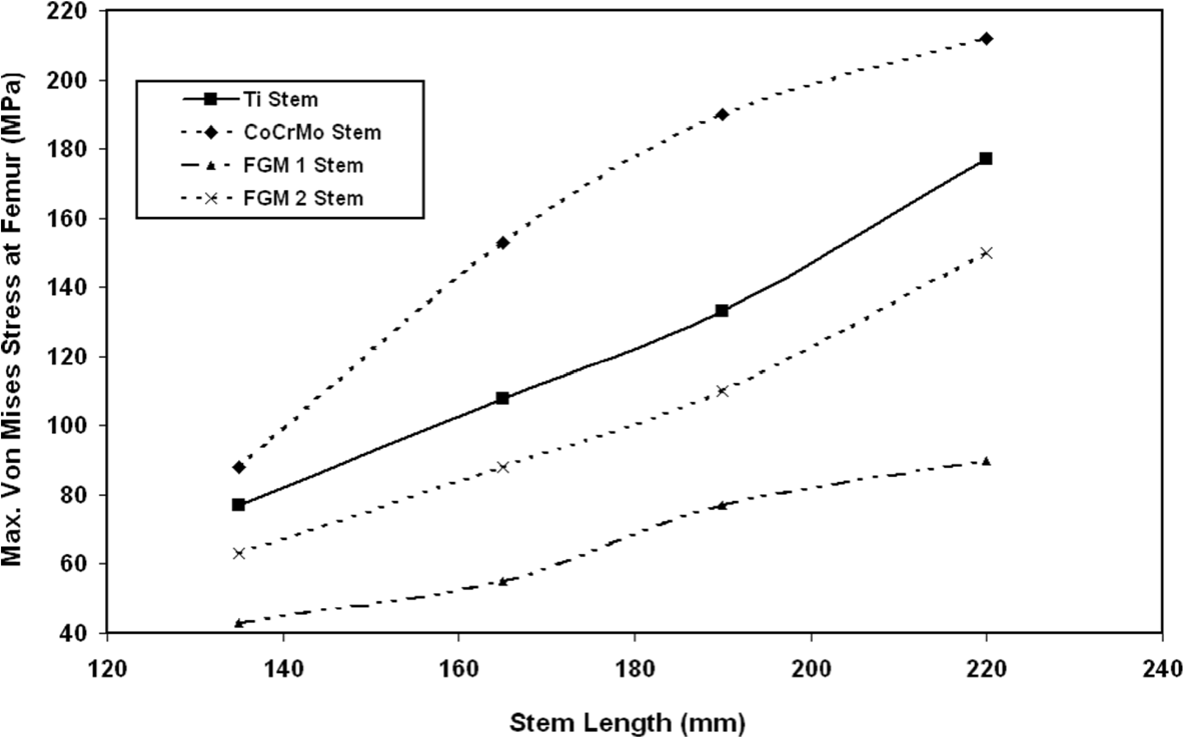
Variation of maximum Von Mises stresses with stem length for FG material stems in comparison with CoCrMo and Ti alloy stems.

The effects of stem stiffness and length on the variation of maximum Von Mises stresses at stem are shown in Figure 15. The results indicate that the maximum Von Mises at stem increase with increasing the stem length. For example, for CoCrMo, the maximum Von Mises stresses at the increase from 88 MPa to 153 MPa, 190 MPa and 212 MPa when using 165 mm, 190 mm and 220 mm CoCrMo stems instead of 135 mm stem. The results show that the maximum Von Mises stress at the stems occurs at the distal end of the stem. This significant increase in the stem Von Mises stresses due to using long stems can be explained due to the increase in the distance between the point of load application (at the femur head) and the hip joint fixation point (at the distal end). The longer distance between the loading point (proximal end) and the hip joint fixation point (distal end) induces larger bending stresses on the whole joint at distal end. Previous results show that the resultant Von Mises stresses at similar Ti alloy stem range from 186 MPa to 253 MPa [7,34]. The present results show that the possible maximum Von Mises stress at stem is 212 MPa, which occurs in case of 220 mm CoCrMo stem. This value is still below the yield strength of Ti ally or CoCrMo stems (800 MPa for Ti alloy and 720 MPa for CoCrMo) [10].

The results of Figure 15 also indicate that the maximum value of Von Mises stresses at stem decrease when using FG material stems instead of CoCrMo and Ti alloy stems at the same testing conditions. For example, for 135 mm stem, the stresses decrease from 88 MPa and 77 MPa to 43 MPa when FG1 stem is used instead of CoCrMo and Ti alloys stems respectively. For 220 mm stem, the stresses at the stem decrease by 57%,49 and 40% when FG1 stem is used instead of CoCrMo, Ti alloy and FG2 stems respectively. The decrease in stem stresses due to using low stiffness FG material can be attributed to the fact that a larger amount of load will be carried by the femur bone specially at the distal end (near fixation point). At this point, the FG stem has E=10 GPa, that is lower than the femur modulus (17 GPa). At case of Ti alloy or CoCrMo stems (that have very high modules compared to femur bone) larger amount of load will be carried by high stiffness stem resulting in a reduction of femur stresses and increases in stem stresses. For all kinds of stems, the maximum Von Mises stresses at stem take place at the distal end. There is no effect for stem length or material on the position of maximum Von Mises stresses at the stem or femur bone and also bone cement.

## Conclusion

From the present FE results, it can be concluded that the maximum Von Mises stresses at the bone cement increase significantly with increasing the stem length while shear stresses decrease. For example, for CoCrMo stem, the maximum Von Mises stress at the bone cement increases by 50% when a stem of 190 mm length is used instead of 135 mm stem length. Similar trends have been also obtained for the Ti alloy, FG1 and FG2 stems with different lengths. The results also indicated that using FG stem (with low stiffness at stem distal end and high stiffness at its proximal end) resulted in a significant reduction in shear stress at the bone cement/stem interface and on the Von Mises stresses at the bone cement compared to CoCrMo and Ti alloy stems. The stresses’ distribution along the bone cement length when using FG material was found to be more uniform along the whole bone cement compared with other stem materials. Also, using FG stem increases the resultant stresses at the femur bone (reduces stress shielding) compared to metallic stems. Accordingly, the authors believe that using FG materials as a stem will be better for reducing the artificial joint stresses and stress shielding effects. This reduction will improve the artificial hip joint short and long term performance.

## Competing interest

The authors certify that there is no actual or potential conflict of interest to declare in relation to this paper.

## Authors’ contributions

FFA-J was involved in the design of study from the clinical point of view, save the artificial stems dimensions as well as helped in the manuscript preparation. HF and OYA participated in the design and analysis of the FE models as well as drafted the manuscript. All the authors have read and approved the final manuscript.
